# Investigation on Bacterial Growth and pH in Milk after the Expiration Date

**DOI:** 10.1155/2023/9982886

**Published:** 2023-10-26

**Authors:** Euphoria Yang, Qing Yang, Brett Troemper, Jianying Zhang

**Affiliations:** ^1^MechanoBiology Laboratory, Department of Orthopaedic Surgery, University of Pittsburgh School of Medicine, 200 Lothrop Street, Pittsburgh, PA 15213, USA; ^2^Franklin Middle School, 1200 Outer Park Drive, Springfield, IL 62704, USA; ^3^Department of Anesthesiology, St. John's Hospital, 800 E Carpenter Street, Springfield, IL 62769, USA

## Abstract

Food waste is a serious national and global problem. Milk is one of the most frequently wasted food items. This study aims to determine how long postexpiration-pasteurized milk may still be safe to consume and what is the relationship between bacterial growth from the milk and time after expiration. The experiments were carried out by incubating milk with agarose gel. The results showed that the bacterial growth was relatively low for at least the first few days after expiration. The more the days passed after the expiration date, the more the bacteria grew from the milk. There was no significant difference in the bacteria colony numbers in the whole milk samples opened either on day 0 or day 5 of expiration. None of the fat-free milk samples collected on the later (1–10) days showed any statistically significant difference in bacterial growth compared to the samples collected on the day after expiration (day 0). The bacterial growth increased with the increasing fat content of the milk. In addition, the rate of bacterial growth from the milk correlated with the acidity of the milk that is measured by pH. No significant sensory changes could be detected in any of the milk samples immediately after opening on the day of expiration or for up to 10 days after expiration when the unopened cartons were kept refrigerated. However, within 24 hours of opening the carton, whole milk that has expired for 6 or more days and 2% milk that has expired for 8 or more days developed a sour taste and mildly pungent smell. This sensory change was accompanied by the formation of lumps and fat-water separation. Fat-free milk samples remained unchanged under the same conditions. The experimental results suggest that whole and 2% skim milk may be safe for consumption up to 5-6 days after expiration. Fat-free milk may be safe for consumption up to 10 days after expiration, possibly longer. This study devised a way to show that milk is still safe for consumption after expiration; it will help reduce food waste.

## 1. Introduction

Food waste is a serious problem. An estimated 40% of food in the United States is wasted annually [1]. Over 90 billion pounds of food are wasted at retail and consumer levels nationally, worth up to $161.6 billion. About 17% of the wasted food is composed of dairy products, such as milk, cheese, and yogurt [1]. In addition to the economic effects, food waste has negative environmental impacts as well. Food waste is the second biggest component of landfills, and landfills are the third largest source of methane emissions. This also affects global warming at an alarming rate [2].

Reducing food waste starts with source control at the retail level [3]. The second tier is feeding hungry people. More than 14% of Americans can be classified as food insecure. Donations can help those in need. The third is feeding animals, which can always benefit us and nature. In order to share food with people or animals, food safety must be ensured [4]. The main reason that food is wasted is that retailers and consumers throw away food when it is expired [1]. Supermarkets and grocery stores routinely take food off the shelves after the expiration date. According to a survey conducted by Harvard and Johns Hopkins Universities, 84% of consumers throw away edible food and the expiration date is reported as the most common cause [1].

Another survey by the National Science Foundation found that 78% of consumers throw out milk and other dairy products once the date on the label has passed [5]. However, expiration dates can be confusing to consumers, especially when they are labeled inconsistently [4]. This is because the FDA does not regulate the date labeling of food products, except for baby formula. The labels “best by” and “use by” often refer to when the food will start declining in quality [6]. In many states, the sell by label is there for managing the inventory in food markets, rather than for alerting when the food will spoil [7]. Twenty-one states prohibit retailers from selling foods, mostly perishables, such as dairy, eggs, meat, and seafood, past their best-before date. For example, in Montana, milk has to be sold within 12 days of pasteurization. This law was put in place to protect the local dairy industry. In Idaho, a neighboring state, milk can be sold for up to 23 days, almost twice as long. In Illinois, there are no rules regarding the specific dates, but generally, dairy manufacturers pick 18–21 days after pasteurization as the expiration date for milk. As such, the laws governing food expiration are arbitrary, and they may be motivated by political reasons, rather than concerns for food safety. Confusion over date labeling on food accounts for nearly 20% of consumer food waste in the US [1]. If the designations of “expiration date,” “best by,” and “enjoy by” do not correlate with the spoilage of the food, then how do people determine if food is still safe to eat after the date? People often use their sense of taste or smell to check if food is spoiled or not, but these methods carry risks because they may get sick from accidentally ingesting spoiled food [8].

There are several ways to test the spoilage of milk, which are also used to ensure proper pasteurization. A traditional way of testing milk is the methylene blue reduction test. Methylene blue is a blue solution that becomes colorless with increased bacterial metabolism. This test is time consuming. An improved version uses an electrode to detect the changes in the current as methylene blue changes color [9]. Acidity increases as milk spoils. Unsoiled milk has a pH of approximately 6.7, and as the pH falls below 5, the protein in milk congeals and precipitates. One study reported that a decrease in pH is associated with rancid and bitter taste in milk [10]. More advanced detection methods include gas sensors to monitor the amount of carbon dioxide produced by bacteria, infrared spectroscopy to detect bacterial metabolic products, and fat or protein counters to detect breaking down of nutrients by bacteria. Lastly, bacteria can be cultured from milk either directly or in serial dilution [11]. Published studies on milk spoilage mostly focused on time after pasteurization, and it is not known whether similar methods can be used on milk after expiration.

It is also unclear if different types of milk spoil at different rates. The only major controlled study on the spoilage of whole and skim milk was inconclusive [8]. The purpose of this experiment was to determine how long postexpiration-pasteurized milk may still be safe to consume. Specifically, we want to know the relationship between bacterial growth from the milk and time after expiration and the change in pH after expiration. We also want to know if different types of milk based on fat content will have significantly different results. We find interest in this investigation because food waste is a serious national and global problem and milk is one of the most frequently wasted food items. If we can devise a way to show that milk is still safe for consumption after expiration, it will help reduce food waste. Therefore, we are interested in investigating the status of different types of milk on various days after expiration. Furthermore, we wish to propose a simple pH-based indicator of milk spoilage.

## 2. Materials and Methods

### 2.1. Materials

Milk products including whole milk, 2% skim, and fat-free half-pint (236 ml) cartons were obtained from a local retail food market (Prairie Farms, Springfield, IL). A total of 11 boxes of each kind of milk, with expiration dates on and up to 10 days prior to the day of experiment, were used. Distilled water (1 gallon bottle) was obtained from Walmart (Bentonville, AR). Agarose (30 g bottle) was obtained from Biomedicals (Santa Ana, CA). Fetal bovine serum (FBS; 100 ml bottle) was obtained from HyClone (Logan, UT). Petri dishes (60 mm diameter), glass beaker, pH strips, hotplate stirrer and magnetic stir bar pipette, and pipette tips were obtained from Fisher Scientific (Pittsburgh, PA). Kitchen scale was obtained from Taylor (Oak Brooks, IL). The M150 biological microscope was obtained from Am Scope (Irvine, CA). The computer was obtained from Lenovo (Quarry Bay, Hong Kong), Image J software was obtained from NIH (Bethesda, Maryland), and Excel software was obtained from Microsoft (Redmond, Washington).

### 2.2. Bacterial Growth Testing in the Milk Cultured with Agarose Gel

All milk samples were kept in a refrigerator at 4°C until the day of the experiment. The workbench surface was wiped with Clorox wipes to sterilize, and the agarose powder (8 g) and 360 ml of distilled water were added to a 500 ml beaker. The agarose solution was boiled on a hotplate with magnetic stirring until a clear solution was obtained. Then, the heat was turned, and 40 ml of fetal bovine serum (FBS) was filtered with 0.2 *μ*m filter and added to the beaker and mixed well when the agarose solution was cooled to 50°C to get an agarose (2%) FBS (10%) solution. The prepared agarose-FBS solution (3 ml) was added to each Petri dish (60 mm diameter), and then 0.3 ml of the milk sample (whole, 2% skim, and fat-free milk on the day of expiration and 1–10 days after expiration) was added to each agarose-FBS solution-containing Petri dish and mixed well to get a homogeneous agarose-FBS-milk solution. The Petri dish was covered with a lid and left at room temperature for 10 min to solidify. Each milk sample was cultured in quadruplicates, producing a total of 132 samples. A new pipette tip was used for each sample. The inoculated Petri dishes were kept in a room with a constant temperature set at 30°C to culture for a total of four days. At 24, 48, 72, and 96 hours after inoculation, a picture was observed and taken of the Petri dish at 10x magnification under the microscope.

### 2.3. Bacterial Colony Analysis by Image J Software

The colony pictures of the bacteria were analyzed by Image J software. The images were converted into 8-bit grayscale, and the threshold was set to accentuate the colonies. Using the analyze particle function to count the colonies, particle size was set to 25–900 pixels and circularity was set at 0-1.

### 2.4. Acidity Analysis of the Milk Samples by the pH Strips

The acidity changes of the milk samples were tested by adding 0.1 ml of each milk sample on the pH paper strip, the color reading was matched against the indicator chart in the pH test strip's packaging, and the result was recorded at different time points.

### 2.5. Sensory Evaluation of the Milk Samples

The sensory evaluation was carried out on two occasions. The first evaluation was performed for all milk samples immediately after opening the carton by adding 20 ml of milk to a clear glass bottle to observe its morphology and color, smell, and taste. The carton was then left at room temperature, and a second sensory evaluation was performed 24 hours later. All evaluations were performed by two experimenters (E.Y. and Q.Y.) blinded to the sample, and the consensus results were recorded.

### 2.6. Statistical Analysis

All experiments were performed in quadruplicates, and the colony numbers were counted by Image J software and recorded in Excel for statistical analysis. Statistical analysis was performed using Student's *t*-test. *p* < 0.05 between the two groups was considered to be significantly different.

## 3. Results

**Figure 1:**
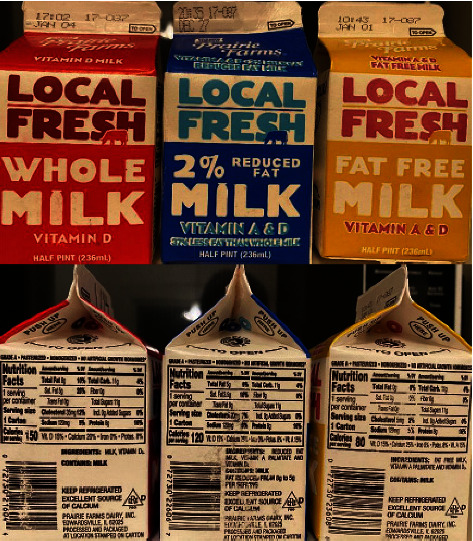
Containers and nutritional labels of the milk samples used in this experiment. Whole, 2% skim “reduced fat”, and fat-free milk from the same manufacturer (Prairie Farms) were used. Note that for each half-pint (236 ml) carton of milk, the total fat content is 8 g, 5 g, and 0 g, for whole, 2%, and fat-free milk, respectively. The other nutritional elements, including sodium, total carbohydrates, total sugars, and protein, are the same for all three types of milk.

**Figure 2:**
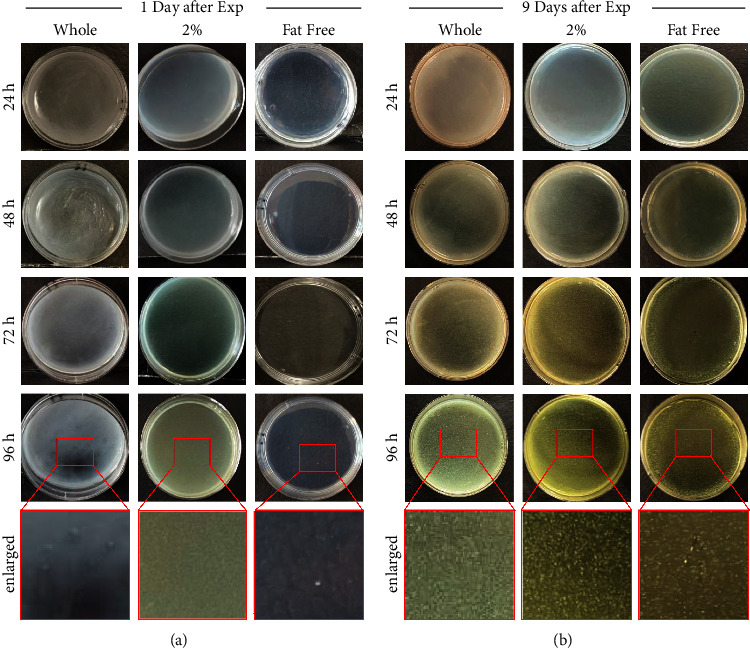
Representative images of Petri dishes at different times after inoculation by milk samples opened at 1 and 9 days after expiration. Very few bacteria colonies were found in all three kinds of milk samples opened one day after expiration and cultured for 4 days (a). Although many bacteria colonies are found in all three milk samples opened 9 days after expiration and cultured for 4 days, very few bacteria colonies are found in the same milk samples opened 9 days after expiration and cultured for 24 hours (b). The bottom row shows an enlarged area with bacteria colonies.

**Figure 3:**
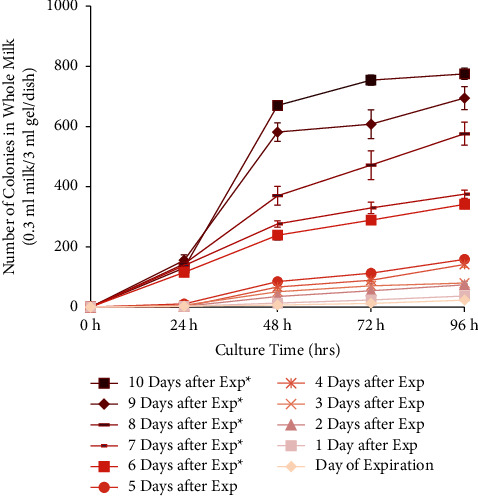
Growth curve of bacteria cultured from whole milk samples opened on the day of and up to 10 days after expiration and 0.3 ml milk cultured in 3 ml of 2% agarose gel with 10% FBS in a 60 mm-diameter Petri dish. Colony numbers were counted at 0, 24, 48, 72, and 96 hours after inoculation and presented as the average of 4 plates. Asterisks indicate significant difference compared to cultures collected on the day of expiration (*t*-test, *p* < 0.05).

**Figure 4:**
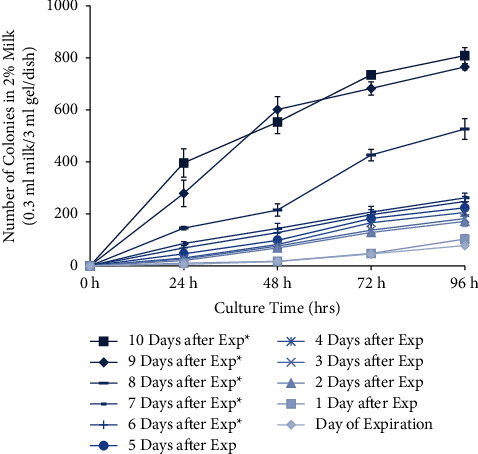
Growth curve of bacteria cultured from 2% skim milk samples opened on the day of and up to 10 days after expiration and 0.3 ml milk cultured in 3 ml of 2% agarose gel with 10% FBS in a 60 mm-diameter Petri dish. Colony numbers were counted at 0, 24, 48, 72, and 96 hours after inoculation and presented as the average of 4 plates. Asterisks indicate significant difference compared to cultures collected on the day of expiration (*t*-test, *p* < 0.05).

**Figure 5:**
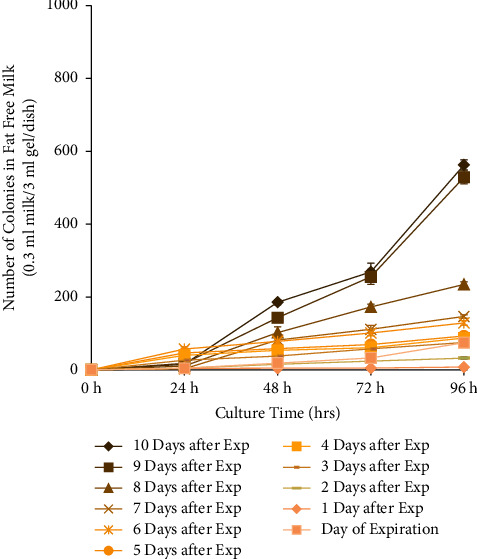
Growth curve of bacteria cultured from fat-free milk samples opened on the day of and up to 10 days after expiration and 0.3 ml milk cultured in 3 ml of 2% agarose gel with 10% FBS in a 60 mm-diameter Petri dish. Colony numbers were counted at 0, 24, 48, 72, and 96 hours after inoculation and presented as the average of 4 plates. None of the samples differed significantly from the day of expiration (*t*-test, *p* ≥ 0.05).

**Figure 6:**
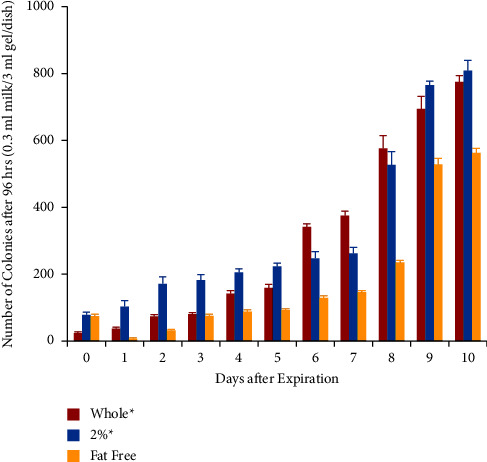
Colony count at 96 hours by milk type and days after expiration when 0.3 ml milk was cultured in 3 ml of 2% agarose gel with 10% FBS in a 60 mm-diameter Petri dish. Colony numbers are the average of 4 samples, and the arrow bars indicate standard deviation. Asterisks indicate significant difference between the groups (^*∗*^*p* < 0.05 compared to fat-free milk).

This experiment tested the bacterial growth and acidity of different types of milk on various days after expiration. Three types of milk were used in this experiment; they were whole, 2% skim, and fat-free, provided by the same dairy manufacturer. According to their nutrition label, the amount of fat contained in each half-pint (236 ml) carton was 8 g, 5 g, and 0 g, for whole, 2% skim, and fat-free milk, respectively (Figure 1). All the other nutritional elements remained the same (Figure 1). All bacteria colony numbers reported here are counted as per Petri dish, which has the standardized size of 60 mm diameter and contained 0.3 ml of milk sample in 3 ml of agarose gel solid medium, totaling 3.3 ml volume. Bacteria grew from all samples tested, and the number of visible colonies per dish increased with the length of culture, reaching several hundred colonies and partial confluency by 96 hours (Figure 2). The growth curve of bacteria cultured from the different types of milk was different. For whole milk, the number of bacterial colonies per dish significantly increased in the samples opened 6 days after expiration. Prior to that, there was no significant difference compared to samples opened on the day of expiration (Figure 3). Similarly, for 2% skim milk, the number of bacterial colonies per dish significantly increased in samples opened 6 days after expiration (Figure 4). In contrast, for fat-free milk, under the same culture conditions, none of the samples collected on the later days showed any statistically significant difference in bacterial growth compared to samples collected on the day after expiration (Figure 5). When comparing between the types of milk, fat-free (562.50 colonies per dish ±13.53 SD; cultured 10 days after expiration for 96 hours) milk had significantly fewer bacteria colonies than whole (775.00 colonies per dish ±19.03 SD) or 2% skim (808.75 colonies per dish ±30.73 SD) milk. There was no statistical difference between whole and 2% skim milk, but skim milk showed a trend of slightly more bacterial growth (Figure 6).

**Figure 7:**
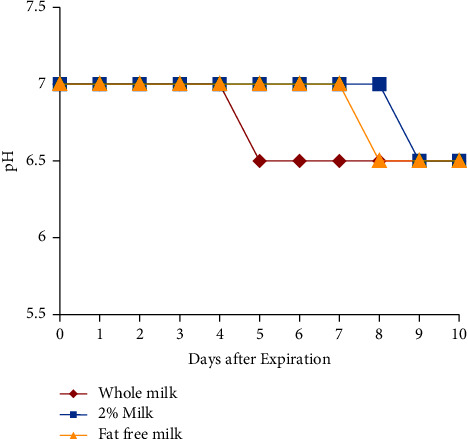
Acidity, as measured by pH paper strips, of milk samples opened on the day of and up to 10 days after expiration.

All samples had pH above 6.5 immediately after opening, although there was a slight decrease in pH from 7 to 6.5 as the days passed after expiration. This drop occurred earlier with the whole milk at 5 days after expiration and then with 2% skim or fat-free milk at 9 and 8 days after expiration, respectively; no definitive assertion can be made due to the lack of granularity in the data (Figure 7).

**Figure 8:**
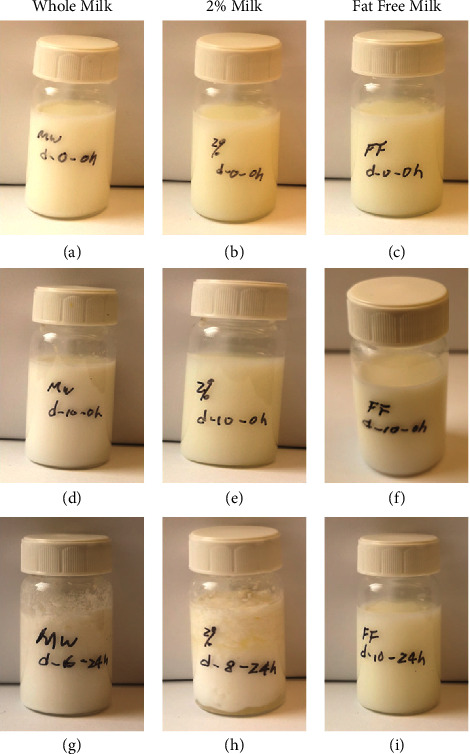
The gross appearance of three types of milk samples opened at different time points after expiration and left at room temperature for 0–24 hours after opening. (a–c) Whole milk (a), 2% milk (b), and fat-free milk (c) immediately after opening on the day of expiration. All three appear as homogeneous white emulsions. (d–f) Whole milk (d), 2% milk (e), and fat-free milk (f) immediately after opening at 10 days after expiration. No visible changes when compared to the day of expiration. (g) Whole milk opened at 6 days after expiration and left open at room temperature for 24 hours appears thickened with lumps. (h) 2% milk sample opened at 8 days after expiration and left open at room temperature for 24 hours appears thickened with lumps. (i) Fat-free milk opened at 10 days after expiration and left at room temperature for 24 hours appears homogeneous.

The sensory evaluation detected no significant change in the color, appearance, smell, or taste of the milk samples immediately after opening on the day of expiration or up to 10 days after expiration (Figures 8(a)–8(f)). All samples of milk appeared white and homogeneous and did not have any pungent smell or sour or bitter taste. To both experimenters who performed the sensory evaluations, all three types of milk appeared, smelled, and tasted the same up to 10 days after expiration as the day of expiration when the unopened milk cartons were kept in the refrigerator. However, differences started to emerge between the samples after the opened cartons were left at room temperature for 24 hours. The whole milk samples opened on day 6 after expiration and left at room temperature for 24 hours after opening thickened with lumps and developed a sour smell and tasted sour as well (Figure 8(g)). These sensory changes were found in 2% milk opened on day 8 after expiration and left at room temperature for 24 hours (Figure 8(h)). On the other hand, fat-free milk samples remained homogeneous with no smell or taste changes even after being left for 24 hours at room temperature following opening (Figure 8(i)). Since the first sensory evaluation performed immediately after opening the milk cartons did not yield any noticeable changes, only the results of the second sensory test performed 24 hours after opening the cartons are presented in summary in Table 1.

## 4. Discussion

This experiment was a head-to-head comparison of bacterial growth and acidity in different kinds of milk after expiration. The results support the experimenter's hypothesis that the bacterial growth increased as more days passed after the expiration dates. The results also support the hypothesis that milk containing fat has more bacteria than fat-free milk. Because the bacterial growth was relatively low initially, the experimental results suggest that whole and 2% skim milk may be safe for consumption up to 5-6 days after expiration. Fat-free milk may be safe for consumption up to 10 days after expiration, possibly longer. Bacterial growth did not increase linearly with fat content, as the number of bacteria colonies growing from the whole and 2% skim milk were similar. This is consistent with previous studies that also failed to show significant differences between these two kinds of milk [12]. The reason may be that a threshold of fat is needed to support bacterial growth, but too much fat may have a suppressive effect.

The acidity of the milk samples did not change significantly during the time period tested. This suggests that pH changes are a late indicator of milk spoilage. Theoretically, a pH-based marker for milk safety is still possible, but the color change needs to be more obvious with the ability to detect subtle shifts in acidity.

The lack of significant changes in pH also corresponded to the lack of sensory changes detected in the milk immediately after opening at up to 10 days after expiration if the cartons were kept refrigerated. Our findings support the idea that smelling or tasting is not a reliable way to detect milk spoilage. Sensory changes happen much later in the process of food spoilage. We found that if the milk cartons were left at room temperature after opening, then within 24 hours, a sour taste and smell would develop, and the milk would start to congeal and separate into fat and liquid for whole milk at 6 days after expiration and 2% milk at 8 days after expiration. But the fat-free milk did not have obvious sensory changes even after being left open for 24 hours at room temperature. This is consistent with our bacteria culture results and confirms that spoilage accelerates at room temperature compared to the refrigerator.

Our results are also consistent with a previously published study by the other researchers on the composition of milk and other dairy products [13]. They found that there were no noticeable changes in fat, protein, acidity, and lactose contents as well as total material count of the sterilized milk before and after the expiration date [13]. However, they only tested the milk products once before and after, and it remained unclear how long postexpiration milk can retain its quality in composition. Also, they only tested whole milk and whether different types of milk (whole, skim, fat-free) spoil at different rates is a point of contention in the debate on food safety and food waste. Our study adds to the existing knowledge by providing a time trajectory of bacterial growth changes in different types of milk after expiration. Our data suggest that whole milk and 2% skim milk may be safe for consumption up to 5-6 days after expiration and fat-free milk is safe for consumption up to 10 days after expiration. Our results can help dairy producers, retailers, and consumers determine when the milk is safe for consumption or donation and decrease the amount of milk discarded simply because of the date labeling. Improving the understanding of milk spoilage can contribute to the extension of safe consumption and reduction in food waste. This can be achieved by policy changes allowing longer time between pasteurization and expiration, by industry initiatives to standardize the nomenclature of expiration date, or by consumer education. In addition, expired dairy products can be used to make soap and cosmetic products [13], further reducing waste.

The main sources of error in this experiment are the way the measurements are made. The number of bacterial colonies detected by the Image J software's particle count function depends on the settings for particle size and circularity. The software can pick up dust under the translucent plate, resulting in a false positive. To maintain internal consistency and ensure precision, the image preprocessing and parameters for the particle count function were kept the same for all samples. In addition, select plates were counted manually to confirm that the particles identified by the software package were indeed colonies.

The acidity testing used a pH paper strip which was not sensitive enough to small changes. Interpretation of the pH values also depended on a visual color scale, which was prone to human error. Another source of error was possible contamination from the environment although all materials and equipment were kept sterile until opening. To mimic the family storage conditions, we did not use a sterile hood or incubator and all milk samples were kept in the home refrigerator and cultured at room temperature, but we cleaned the work surface with antibacterial wipes prior to starting the experiment. All Petri dishes were covered for the duration of culture in an effort to prevent contamination. In the future, we will repeat the experiment under sterile culture conditions. For the current study, we used agarose gel to culture the milk samples which is not able to reliably determine the types or species of bacteria. Published work shows that bacteria commonly found in milk and dairy products include *Bacillus, Streptococcus, Lactococcus, Lactobacilli, Bifidobacteria, Paenibacillus, Enterococcus, Pseudomonas,* and *Enterobacter* [11, 14]. Although not all bacterial species are pathogenic, bacterial growth can contribute to changes in the properties of milk through both spoilage and fermentation processes. In future studies, we plan to use specific agar gels suitable for the differential cultures of various types of bacteria, so we can select pathogenic or harmful bacteria. We can use polymerase chain reaction to characterize the type and amount of bacteria present in the milk. We can also use commercial coliform testing kits and check for the presence of total coliform, fecal coliform, and *E. coli* bacteria. We will increase the number of days after expiration to test the samples, especially for fat-free milk. Using a pH probe that can give more granular readings would help. Lastly, it would be interesting to investigate changes in the sugar content of milk and the rate of spoilage, for example, by testing chocolate milk which has a large amount of added sugar.

## 5. Conclusion

In summary, this experiment demonstrated that milk can remain relatively low in bacterial growth several days after the expiration date. There was no significant difference in the bacteria colony numbers in the fat-free milk samples on the expiration day and 10 days after expiration. Similarly, the number of bacteria in the whole milk on the expiration day (day-0) is the same as that at day 5 after expiration, indicating that the milk is still safe after 5 days of expiration. This information can help retailers and consumers determine when milk is safe for consumption or donation and decrease the amount of milk discarded simply because of the date labeling. A simple pH-based indicator may be a useful tool for milk spoilage.

## Figures and Tables

**Table 1:** Sensory test of three milk samples left open for 24 hours.

Milk type	Whole milk					2% milk					Fat-free milk				
Days after exp.	pH	Color	Smell	Taste	Appearance	pH	Color	Smell	Taste	Appearance	pH	Color	Smell	Taste	Appearance
0	7	White			Homogeneous	7	White			Homogeneous	7	White			Homogeneous
1	7	White			Homogeneous	7	White			Homogeneous	7	White			Homogeneous
2	7	White			Homogeneous	7	White			Homogeneous	7	White			Homogeneous
3	7	White			Homogeneous	7	White			Homogeneous	7	White			Homogeneous
4	7	White			Homogeneous	7	White			Homogeneous	7	White			Homogeneous
5	6.5	White			Homogeneous	7	White			Homogeneous	7	White			Homogeneous
6	6.5	White			Small clot+	7	White			Homogeneous	7	White			Homogeneous
7	6.5	White	sour+		Small clot++	7	White			Homogeneous	7	White			Homogeneous
8	6.5	White	sour+	sour+	Large clot+, liquid+	7	White			Small clot+, liquid+	6.5	White			Homogeneous
9	6.5	White	sour++	sour+	Large clot++, liquid+	6.5	White	sour+		Small clot+, liquid+	6.5	White			Homogeneous
10	6	Light yellow	sour+++	sour++	Large clot+++, liquid+	6.5	White	sour++	sour+	Small clot++, liquid++	6.5	White			Homogeneous

*Note.* All samples were kept in a refrigerator (4°C) until opening. The sensory evaluation was tested for each sample which was opened on the days (0–10) after expiration and left at room temperature for 24 hours after opening. Of note, there were no sensory or appearance changes in any of the samples immediately after opening.

## Data Availability

The data used to support the findings of the study are available on request from the corresponding author.
